# Bioactivity of Different Chemotypes of Oregano Essential Oil against the Blowfly *Calliphora vomitoria* Vector of Foodborne Pathogens

**DOI:** 10.3390/insects12010052

**Published:** 2021-01-11

**Authors:** Stefano Bedini, Priscilla Farina, Edoardo Napoli, Guido Flamini, Roberta Ascrizzi, Antonella Verzera, Barbara Conti, Lucia Zappalà

**Affiliations:** 1 Department of Agriculture, Food and Environment, University of Pisa, Via del Borghetto 80, 56126 Pisa, Italy; stefano.bedini@unipi.it (S.B.); priscilla.farina@phd.unipi.it (P.F.); 2 Institute of Biomolecular Chemistry-National Research Council (ICB-CNR), Via P. Gaifami 18, 95126 Catania, Italy; edoardo.napoli@icb.cnr.it; 3 Department of Pharmacy, University of Pisa, Via Bonanno 6, 56126 Pisa, Italy; guido.flamini@unipi.it (G.F.); roberta.ascrizzi@gmail.com (R.A.); 4 Department of Veterinary Science, University of Messina, SS. Annunziata, 98168 Messina, Italy; antonella.verzera@unime.it; 5 Department of Agriculture, Food and Environment, University of Catania, Via Santa Sofia, 100, 95123 Catania, Italy; lucia.zappala@unict.it

**Keywords:** botanical insecticides, chemotypes, essential oils, repellent, Diptera, foodborne disease, *Origanum vulgare*

## Abstract

**Simple Summary:**

*Calliphora vomitoria* L. is a very common synanthropic blowfly. Since it is attracted by human food, it plays a main role in the transmission of foodborne diseases. Among aromatic plant essential oils (EOs), those of spices are the most suitable to protect food from insect pests. In the present work, we determined the bioactivity of three oregano EOs against *C. vomitoria*. The chemical analyses showed that the EOs belonged to three chemotypes, one with a prevalence of carvacrol and two with a prevalence of thymol. The bioassays showed that the bioactivity of the EOs significantly varies among chemotypes, with the thymol chemotype showing an overall higher efficacy compared to the carvacrol one.

**Abstract:**

Blowflies play a substantial role as vectors of microorganisms, including human pathogens. The control of these insect pests is an important aspect of the prevention of foodborne diseases, which represent a significant public health threat worldwide. Among aromatic plants, spices essential oils (EOs) are the most suitable to protect food from insect pests. In this study, we determined the chemical composition of three oregano EOs and assessed their toxicity and deterrence to oviposition against the blowfly *Calliphora vomitoria* L. The chemical analyses showed that the EOs belonged to three chemotypes: one with a prevalence of carvacrol, the carvacrol chemotype (CC; carvacrol, 81.5%), and two with a prevalence of thymol, the thymol/*p*-cymene and thymol/γ-terpinene chemotypes (TCC and TTC; thymol, 43.8, and 36.7%, respectively). The bioassays showed that although all the three EOs chemotypes are able to exert a toxic activity against *C. vomitoria* adults (LD_50_ from 0.14 to 0.31 μL insect^−1^) and eggs (LC_50_ from 0.008 to 0.038 μL cm^−2^) as well as deter the oviposition (Oviposition Activity Index, OAI, from 0.40 ± 0.04 to 0.87 ± 0.02), the bioactivity of oregano EOs significantly varies among the chemotypes, with the thymol-rich EOs (TCC and TTC) overall demonstrating more effectiveness than the carvacrol-rich (CC) EO.

## 1. Introduction

Foodborne diseases are a significant public health threat and a major cause of morbidity worldwide [1]. Every year, both in developing and industrialized countries, a high percentage of people are subjected to illnesses caused by food bacterial contamination [2]. Among insect pests, blowflies play a substantial role as vectors of microorganisms, including human pathogens [3]. As a result of their feeding and reproductive behavior, blowflies come into contact with the numerous microorganisms that thrive in waste, decaying tissues, and excrements [4]. As a consequence, blowflies can act as reservoirs for the transmission of pathogens such as *Salmonella typhimurium*, *Taenia* spp., *Entamoeba coli*, *Giardia duodenalis*, and *Mycobacterium avium* subspecies *paratuberculosis* that can survive on the body or in the digestive system of the flies [2,3]. Then, such pathogens may be transmitted to food by the contact of the insects’ legs and hairs but also by their saliva, digestive enzymes, and feces when they land on food surfaces [3,5].

The bluebottle fly *Calliphora vomitoria* (L.) (Diptera: Calliphoridae) is a common synanthropic blowfly present in most areas of the world [6]. *C. vomitoria* has been shown to act as a vector of pathogenic microorganisms [2], and its maggots may cause human and animal cutaneous myiasis [7,8].

Currently, the control of blowflies mainly relies on synthetic insecticides [9,10]. However, their use is often not allowed in the presence of food. In addition, the negative effects of synthetic pesticides and repellents on humans, animals, and the environment [11,12,13], as well as the insurgence of insect resistance due to the repeated administration of the synthetic agents [14,15], have raised an increased interest for new eco-friendly and safe control tools [16,17]. On the contrary, the essential oils (EOs) of aromatic plants are characterized by low toxicity toward mammalians and high biodegradability [18]. Moreover, EOs of aromatic plant species, commonly used as food spices, have the advantage of being legally allowed in food and to have a smell profile already accepted by consumers [19,20,21,22,23].

Oregano, *Origanum vulgare* L. (Labiatae), is an aromatic plant native to Western and Southwestern Eurasia and the Mediterranean sub-region [24]. It is widely consumed worldwide as a fresh culinary herb or dried spice, and it is listed as “Generally Recognized As Safe” [25]. Oregano is commonly used as a preservative and flavoring agent in several foodstuffs, alcoholic beverages, cosmetics, and soaps [26,27]. Depending on its chemical composition, oregano EO is classified into two main chemotypes: carvacrol and thymol type, based on the relative content of the two phenols [28,29].

Oregano EO shows a strong antimicrobial and antifungal activity toward human, plant, and foodborne disease pathogens [30,31,32,33,34,35,36,37], and it has been tested, with promising results, for the control of several Coleoptera, Diptera, and Lepidoptera species [37,38,39,40,41].

Despite the evidence of oregano EO bioactivity against insect pests, to the best of our knowledge, no information is available on its bioactivity against blowflies. Therefore, continuing our studies on the bioactivity of spices EOs against blowflies [19,20,42], we herein aim to assess the toxicity and the oviposition deterrence of oregano EOs against the disease-carrying blowfly *C. vomitoria*. Moreover, since EOs’ bioactivity mainly depends on their chemical composition, we evaluate the differences in the bioactivity of three oregano EO chemotypes.

## 2. Materials and Methods

### 2.1. Plant Material

Oregano carvacrol chemotype (CC, *O. vulgare* L.) was collected in the province of Almería (Andalusia, Spain). Oregano thymol/*p*-cymene chemotype (TCC, *O. vulgare* ssp. *hirtum* (Link) Ietsw. was collected in the province of Messina (Sicily, Italy). Oregano thymol/γ-terpinene chemotype (TTC, *O. vulgare* L.) EO was purchased from Altanatura^®^, Inalme S.r.l., (Catania, Sicily, Italy).

### 2.2. Essential Oils Extraction and Chemical Analysis

Inflorescences and leaves of the CC and TCC oregano (100 g) were dried at room temperature in the shadow, until constant weight, and then subjected to hydrodistillation. The hydrodistillation was performed according to the current European Pharmacopoeia [43] until there was no significant increase in the volume of oil (3 h). The oil was dried over anhydrous sodium sulfate (Na_2_SO_4_) and stored under N_2_ in a sealed vial until required.

GC/EI-MS (Gas Chromatography/Electron Impact-Mass Spectrometry) analyses were performed with a Varian CP-3800 apparatus (Agilent Technologies Inc., Santa Clara, CA, USA) equipped with a DB-5 capillary column (30 m × 0.25 mm i.d., film thickness 0.25 μm) and a Varian Saturn 2000 ion-trap mass detector (Agilent Technologies Inc., Santa Clara, CA, USA). The operating conditions were as described in Giuliani et al. [44]. The oven temperature was programmed rising from 60 to 240 °C at 3 °C /min; injector temperature 220 °C; transfer-line temperature 240 °C; carrier gas He (1 mL/min). For each EO, the injection volume was 1 μL, after dilution in *n*-hexane HPLC grade at 5% *v*/*v*. Percentages of compounds were determined from their peak areas in their GC profiles. The identification of constituents was based on the comparison of their retention times with those of the authentic samples (when available), comparing their linear retention indices relative to the series of C9–C25 *n*-hydrocarbons. Computer matching was also used against a commercial [45] and a laboratory-developed mass spectra library, which was built up from pure substances and components of commercial essential oils of known composition and MS literature data [46].

### 2.3. Calliphora vomitoria Rearing

Flies rearing was performed according to Bedini et al. [19], with minor changes. Larvae of *C. vomitoria* were purchased from a commercial supplier (Fish Company Arco Sport, Cascina (PI), Italy). Larvae were fed with minced beef liver and maintained under laboratory conditions (23 °C, 60–70% relative humidity, natural photoperiod) until pupation. 

After the identification, adult flies were put in a 47.50 × 47.50 × 93.0 cm cage (BugDorm-4M4590DH Specimen Handling Cage, MegaView Science Co., Ltd., Taichung, Taiwan) in knitted mesh and polyester. Flies were fed with a solid diet (sugar and yeast 4:1) and water *ad libitum*. The sugar–yeast diet was previously shown to be successful in providing the protein amounts necessary to stimulate the oviposition in Calliphoridae flies [47,48]. The adult *C. vomitoria* population was maintained under laboratory conditions (23 °C, 60–70% relative humidity, natural photoperiod).

### 2.4. Toxicity Bioassays

Unsexed adults (7–10 days old) were treated by topical application of the EOs with a Burkard micro-applicator (Burkard Scientific Ltd., Uxbridge, UK), using a 1 mL syringe. Then, 2 μL of 0.0 (control), 1.00, 2.50, 5.0, 7.50, 10.0, 20.0, 30.0, and 40.0% ethanol (EtOH) solutions of the EOs, corresponding to 0.0 (control), 0.02, 0.05, 0.10, 0.15, 0.20, 0.40, 0.60, and 0.80 µL EO insect^−1^ were applied on the thorax of adults (20 individuals per EO concentration). To ease the application of the solutions, the flies were put in a Falcon tube with a netted cap and anesthetized at −18 °C for 3 min. Then, treated insects (20 individuals per cage) were maintained in Plexiglas cages (20 cm of diameter, 30 cm in length) with sugar and water *ad libitum* under laboratory conditions (23 °C, 60–70% RH, natural photoperiod). For each EO concentration, three replicates were performed. Flies mortality was checked after 24 h, and values were corrected using Abbott’s formula [49].

The toxicity evaluation of oregano EOs against *C. vomitoria* eggs was performed according to Bedini et al. [42], with minor changes. Eggs were obtained from adult females supplied with warm minced beef to stimulate the oviposition. Then, 4.5 × 4.5 cm squares of filter paper (surface 20.2 cm^2^) were treated with 100 μL of 0.0 (control), 0.1, 0.2, 0.5, 0.75, and 1.0%. EtOH solution of oregano EO, corresponding to 0.0 (control), 0.005, 0.010, 0.025, 0.037, and 0.050 μL EO cm^−2^. The ethanol was evaporated from the paper under airflow for 3–5 min before placing the eggs, and the squares of filter paper were wetted with 380 μL of water and placed in glass Petri dishes (10 cm of diameter). Finally, fifty newly laid eggs (0–12 h old) were collected and arranged, using a wet fine brush, on the surface of each of the paper squares. The eggs were incubated in Petri dishes kept in a climatic chamber (KW Srl, Siena (SI), Italy), in the dark, at 25 °C. Eggs hatching was recorded every 24 h for three days (24, 48, and 72 h), counting the egg’s chorions under a dissecting microscope (Nikon SMZ1500, Nikon Instruments Inc., Tokyo, Japan). At each check, the squares of filter paper were re-wetted with 380 μL of water. For each EO concentration, five replicates were performed. Eggs mortality values were corrected using Abbott’s formula [49].

### 2.5. Oviposition Deterrence Bioassay

The EOs’ protective effect on the meat was evaluated by oviposition deterrence assays performed according to Bedini et al. [19,42], with minor changes. One hundred and fifty unsexed adults of *C. vomitoria*, 10-14 days old, were placed into 47.50 × 47.50 × 93.0 cm cages (BugDorm-4M4590DH). Flies were fed with sugar and yeast (4:1) for the entire duration of the test. To stimulate the oviposition, the cages were provided with plastic cylinders (3.50 cm of diameter, 5.0 cm in height, surface area 9.61 cm^2^) each containing 8 g of minced pork, added with water (20% *w*/*w*) to avoid desiccation. The meat surface was flattened and sprayed with 150 µL of oregano EOs solutions in EtOH, using a glass nebulizer. Tested concentrations of the three EOs were 0.0 (control), 0.5, 1.0, and 2.0%, corresponding to 0.0 (control), 0.08, 0.16, and 0.32 μL EO cm^−2^. Four dishes, containing the four cylinders, each treated with one of the four EOs concentrations, were placed at each of the four inner corners of the cage, at about 5 cm from the edges. To prevent position biases, meat samples were placed in the same order in each corner of the cage. Cages were put under fluorescent lamps (14,000 lux) to provide even lighting and maintained at about 23 °C and 75% RH. A beaker (covered by a net) containing 500 mL of water was put in each cage to maintain the humidity. The whole experiment was replicated three times. Laid eggs were counted 24 h after the beginning of the assay (mean number of eggs laid per cage, 27,010 ± 8106), using the piece counter function of an analytical balance (KERN ABS-N, Kern & Sohn, Balingen, Germany).

The EOs’ protective effect was expressed as Oviposition Activity Index (OAI), which was calculated using the following formula:

OAI = [(NC − NT)/(NT + NC)]

where NC is the total number of eggs laid on the Control meat (treated with EtOH only) and NT is the total number of eggs laid on the treated meat [50].

### 2.6. Data Analyses

The median lethal dose (LD_50_) of the EOs against *C. vomitoria* adults and median lethal concentration (LC_50_) of the EOs against *C. vomitoria* eggs were calculated by Log-probit regression [51]. Log-probit regression curves were compared by relative median potency (RMP) after having checked for their parallelism [52]. Significant differences among the LD/LC values of the EO chemotypes were determined by estimating the confidence intervals of RMP. The differences were considered statistically significant when values in the 95% confidence interval of relative median potency analyses were ≠ 1.0. To confirm the Probit results (and in the meantime to provide a more synthetic output of the results), bioassays data were also processed by one-way between-groups univariate analysis of covariance (ANCOVA), with the EOs as a fixed factor and the dose/concentration as a covariate to control its effects in the model. The mean response for each factor (EOs), adjusted for the dose/concentration, was reported as estimated marginal (EM) means, and significant differences among them were determined by *post hoc* comparisons using Bonferroni corrections for multiple comparisons [20,21,53,54]. The EOs protective effect data were processed by the Kruskal–Wallis test with the OAI as a factor. Means were separated by Dunn–Bonferroni pairwise comparisons [55]. Statistics were performed by SPSS 22.0 software (IBM SPSS Statistics, Armonk, North Castle, NY, USA).

## 3. Results

### 3.1. Chemical Analysis

The compositions of the three oregano EOs are reported in Table 1 (compounds over 0.1%). The GC-EIMS analysis of the EOs identified 32, 39, and 15 compounds in the TCC, TTC, and CC oregano chemotypes, respectively. These compounds correspond, respectively, to 99.5, 98.4, and 100.0% of the total compositions. In particular, the main constituents of the TCC and TTC oregano EOs were as follows: *p*-cymene, 18.4 and 9.3%; γ-terpinene, 11.4 and 19.7%; thymol, 43.8 and 36.7%, respectively. The CC oregano EO composition was dominated by carvacrol (81.5%), followed by *p*-cymene (8.0%). Carvacrol was also detected in TCC and TTC oregano chemotypes, although in considerably lower relative abundances (6.0 and 0.9%, respectively).

### 3.2. Toxicity Bioassays

**Figure 1:**
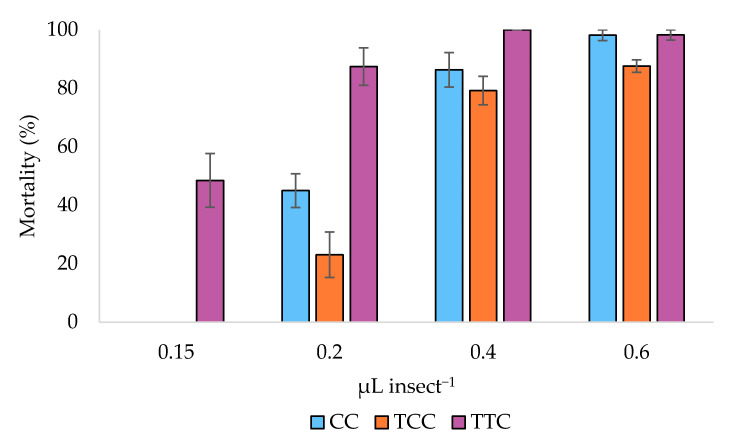
Adulticidal activity of the essential oils of the carvacrol (CC), thymol/*p*-cymene (TCC), and thymol/γ-terpinene (TTC) oregano chemotypes essential oils. The histograms represent the mean mortality percentage (%). Bars represent standard errors.

The three oregano chemotypes EOs showed a clear toxic action by direct contact when administered on the thorax of adult flies (Figure 1).

ANCOVA indicated statistically significant differences among the EOs chemotypes (*F*_2,26_ = 7.742, *p* = 0.002). Estimated marginal (EM) means showed that the most effective chemotype was the TTC, while the TCC was the less effective (Table 2).

**Figure 2:**
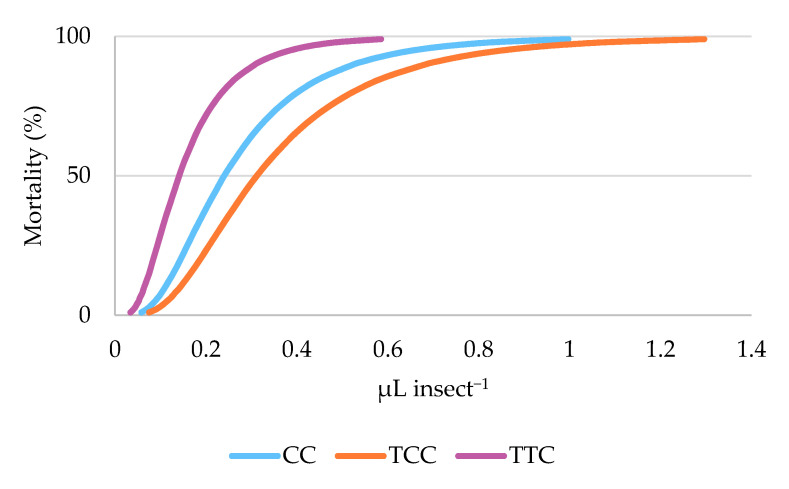
Dose–effect relationship of the mortality (mortality %) of adults of the synanthropic fly *Calliphora vomitoria* exposed by contact to carvacrol (CC), thymol/*p*-cymene (TCC), and thymol/γ-terpinene (TTC) oregano chemotypes essential oils as predicted by the Probit model.

In particular, the post hoc tests indicated a significant difference between TTC and TCC (Bonferroni pairwise comparison, *p* = 0.002), but no differences between TCC and CC nor between TTC and CC were evidenced (Bonferroni pairwise comparison, *p* = 0.269 and 0.150, respectively) (Table 2). Consistently, LD_50_ of the three EOs chemotypes, calculated by Probit analysis, were 0.141, 0.240, and 0.312 μL insect^−1^ for TTC, CC, and TCC, respectively (Table 3, Figure 2).

The RMP analysis showed significant differences in toxicity between TTC and CC (RMP = 0.586, CI = 0.442–0.736), between CC and TCC (RMP = 0.770, CI = 0.633–0.917), and between TTC and TCC (RMP = 0.451, CI = 0.322–0.586) against adult *C. vomitoria* flies.

**Figure 3:**
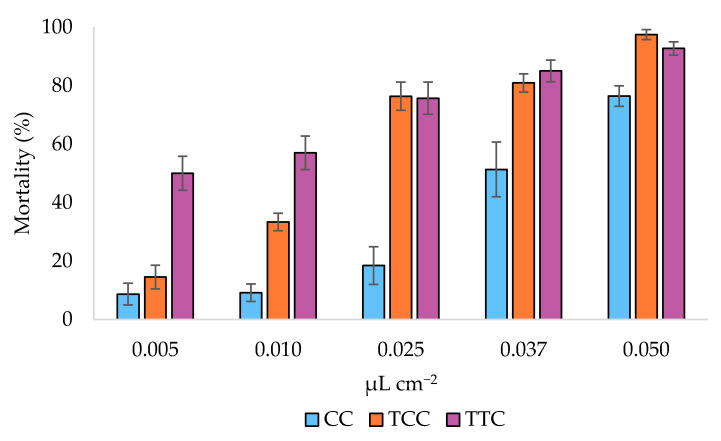
Ovicidal activity of the essential oils of the carvacrol (CC), thymol/*p*-cymene (TCC), and thymol/γ-terpinene (TTC) oregano chemotypes. The histograms represent the mean mortality percentage (%). Bars represent standard errors.

Toxicity tests on *C. vomitoria* eggs showed the different toxicity of the EOs with the three oregano chemotypes (ANCOVA, *F*_2,43_ = 79.649, *p* < 0.001) (Figure 3).

In detail, the estimated marginal (EM) means (Table 4) showed that the most effective chemotype was the TTC, while the CC was the least effective one.

**Figure 4:**
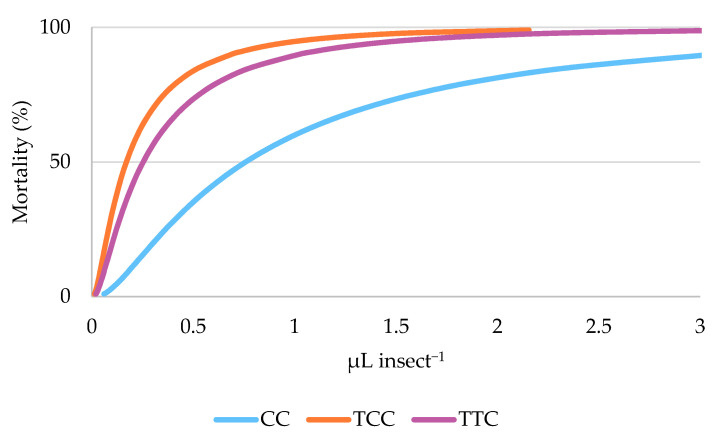
Dose–effect relationship of the mortality (mortality %) of *Calliphora vomitoria* eggs exposed by contact to carvacrol (CC), thymol/*p*-cymene (TCC), and thymol/γ-terpinene (TTC) oregano chemotypes essential oils as predicted by the Probit model.

The EM means post hoc tests indicated that the differences among the ovicidal activity of the three EO chemotypes are significant (Bonferroni pairwise comparison, *p* < 0.001). LC_50_ of the three EOs chemotypes were 0.038, 0.008, and 0.013 μL cm^−2^ for CC, TCC, and TTC chemotypes, respectively (Table 5, Figure 4).

The RMP analysis showed that the CC chemotype EO was significantly less effective in killing *C. vomitoria* eggs than the other two oregano chemotypes EOs (TTC vs. CC RMP = 0.333 (0.093–0.702); TCC vs. CC RMP = 0.225 (0.049–0.521).

### 3.3. Oviposition Deterrence Bioassay

**Figure 5:**
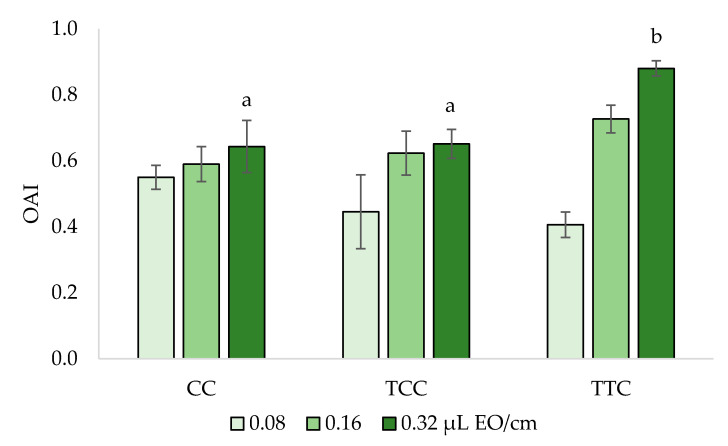
Oviposition deterrence against *Calliphora vomitoria* of the essential oils of carvacrol (CC), thymol/*p*-cymene (TCC), and thymol/γ-terpinene (TTC) oregano chemotypes. Histograms represent the mean values of the Oviposition Activity Index (OAI). Bars represent standard errors. For each concentration, different letters indicate a significant difference among EOs by Dunn–Bonferroni pairwise comparison (*p* < 0.05).

Oviposition deterrence assays indicated that all the EOs are able to strongly affect the oviposition behavior of *C. vomitoria* females. Mean OAI values ranged from 0.41 to 0.88 (Figure 5), depending on the concentration and the EO chemotype.

No significant difference among the EOs chemotypes was observed at 0.08 and 0.16 μL cm^−2^. On the contrary, at the highest concentration tested (0.32 μL cm^−2^), the Kruskal–Wallis test showed a significant difference among the EO chemotypes (*χ*^2^ = 6.033; df = 2; *p* = 0.049). The Dunn–Bonferroni pairwise comparisons of the OAI values indicated that the protective effect of the TTC was significantly higher than the one of TCC and CC oregano EOs (TTC vs. TCC, *p* = 0.019; TTC vs. CC, *p* = 0.045).

## 4. Discussion

Insects vectors of microorganisms are responsible for the loss and spoilage of a huge quantity of food and the spread of foodborne disease [1]. Spices EOs are widely used as a food ingredient for their aroma and preservative properties [56], and their toxic and repellent activity against food insect pests is reported in the literature [19,20,22,57], but very little is known about their use against synanthropic flies. Here, for the first time, we tested three oregano EOs chemotypes as insecticides and repellents against the blowfly *C. vomitoria*, which is a synanthropic fly vector of foodborne diseases.

The chemical analyses of the three oregano EO chemotypes tested in this work showed that phenols are the main components of the EOs. According to the classification by Napoli and Ruberto [58], two of the tested EOs (TCC and TTC EOs) belong to the thymol chemotype, while the CC EO belongs to the carvacrol chemotype. Thymol and carvacrol are reported as the major components of oregano EOs [59,60,61]. Vokou et al. [62] analyzed the chemical composition of the EOs extracted from oregano samples collected in twenty-three localities in Greece. Their results showed that despite the presence of EOs composed for over 90% by thymol or carvacrol, all the intermediate combinations between the two phenols can be found. On the contrary, thirty-six samples of *O. vulgare* collected from more than twenty localities in Turkey showed a prevalence of carvacrol in their compositions (carvacrol from 23.4 to 78.7%) [63,64]. The relationship between the increase of the content of one phenol to the decrease of the other one indicates that there is a biosynthetic correlation between thymol and carvacrol [65].

Regarding the lesser components, Kokkini et al. [66] suggested that differences in the contents could be related to the harvesting season. In fact, oregano samples collected in autumn in three distinct geographic areas of Greece had a lower amount of γ-terpinene and a higher amount of *p*-cymene compared to the summer ones.

Regarding our results, the bioassays showed higher toxicity by contact against *C. vomitoria* adults of the TTC, which contains thymol and γ-terpinene as main components (36.70 and 19.70%, respectively). A traditional application of oregano against flies is known in Albania, where dried grounded oregano leaves are traditionally used to keep flies away from houses and facilities where foods are processed [67]. Actually, Xie et al. [41] observed the toxicity of *O. vulgare* EO against the housefly *Musca domestica* L. (Diptera: Muscidae). In agreement with our observations, Karpouthsis et al. [68] showed that the *O. vulgare* subsp. *hirtum* and *Coridothymus capitatus* (L.) EOs containing higher percentages of carvacrol (74.56 and 81.46%) were less effective than the thymol-rich EO of *Satureja thymbra* L. (Lamiaceae) as insecticide agents against *Drosophila melanogaster* (Diptera: Drosophilidae) larvae.

In previous studies focused on the application of EOs extracted from aromatic plants, which are generally used as spices, Bedini et al. [20] observed LD_50_ by contact against *C. vomitoria* as 0.44, 1.10, and 1.97 μL insect^−1^ respectively for garlic (*Allium sativum* L.), rosemary (*Rosmarinus officinalis* L.), and sage (*Salvia officinalis* L.) EOs, with significantly higher toxicity exerted by the garlic one. Similarly, tarragon (*Artemisia dracunculus* L.) EOs showed clear toxicity against *C. vomitoria* with an LD_50_ of 0.49 μL EO insect^−1^ [19]. The three oregano EOs tested in this research showed higher toxicity than the garlic, rosemary, and sage EOs, with the TTC (0.14 μL insect^−1^) that was about four times more toxic than the *A. sativum* EO (0.44 μL insect^−1^) but very close to that of the tarragon. In line with the toxicity toward adults, ovicidal tests showed that the two oregano thymol-type EOs (TCC and TTC) were significantly more effective than the carvacrol one.

The higher effectiveness of the thymol-type EOs was also confirmed by the oviposition deterrence tests. All EOs were able to strongly affect the oviposition behavior of *C. vomitoria* females, but after 24 h, the protection effect of 0.32 µL cm^−2^ of the TTC EO was significantly higher than the other two EOs. The complete protection of the meat from *C. vomitoria,* up to 24 h, oviposition was also obtained by *A. sativum* EO, but at a much higher concentration (1.25 µL EO cm^−2^) [20], while a stronger protective effect was observed for the spice *A. dracunculus* EO that was able to completely deter *C. vomitoria* oviposition from 0.05 µL EO cm^−2^ [19].

One of the main mechanisms at the bases of the toxicity of EOs against blowflies is the inhibition of acetylcholinesterase [19,42], which is an important enzyme in neuronal and neuromuscular communication, whose only difference between its insect and mammalian counterparts is a single residue, making AChE an insect-selective target for newly developed insecticides [40]. For this reason, EOs may represent an effective specific ingredient in the formulation of pesticides and repellents that is effective against the target insect pest and safe for humans and suitable to be used for food protection as well. Although their strong smell may interfere with the sensory quality of food, EOs extracted from spices, thanks to their established acceptance as ingredients in food, may overcome such withdraw.

## 5. Conclusions

Our results indicate that oregano EOs may represent an effective tool for the control of blowflies. Our data showed that the EOs bioactivity against *C. vomitoria* varies depending on the chemical composition of the EO chemotypes. Thus, an extensive EOs chemotyping, coupled with the specific bioassays, may lead to defining standards of EO chemical compositions that are able to ensure the constant and reliable activity needed for the formulation of insecticides or repellents based on spice EOs to be used for the protection of food.

## Figures and Tables

**Table 1:** Chemical compositions (compounds > 0.1%) of the essential oils hydrodistilled from the aerial parts of the three *Origanum vulgare* accessions.

Compounds	l.r.i. ^a^	Relative Abundance (%)		
		TCC	TTC	CC
(*E*)-Hexenal	855	- ^b^	0.1	-
Tricyclene	928	1.1	1.8	-
Cumene	929	0.8	0.8	-
α-Thujene	931	-	-	0.4
α-Pinene	941	0.1	0.1	0.1
1-Octen-3-ol	980	0.2	0.2	1.8
3-Octanone	987	0.1	-	0.8
β-Myrcene	993	2.1	2.6	1.1
α-Phellandrene	1005	0.2	0.4	-
*iso*-Sylvestrene	1019	0.1	0.1	-
α-Terpinene	1020	2.1	3.3	0.8
*p*-Cymene	1027	18.4	9.3	8.0
Limonene	1032	0.7	0.6	-
Eucalyptol	1034	-	-	0.1
(*Z*)-β-Ocimene	1042	1.5	3.2	-
(*E*)-β-Ocimene	1052	0.3	0.7	-
γ-Terpinene	1062	11.4	19.7	2.4
*cis*-Sabinene hydrate	1070	-	0.2	-
Terpinolene	1088	0.2	0.1	-
Linalool	1101	0.6	1.2	0.3
Borneol	1165	0.2	0.2	-
Terpinen-4-ol	1178	0.7	1.0	0.2
α-Terpineol	1189	0.1	0.2	-
Thymol methyl ether	1235	1.9	3.8	-
Carvacrol methyl ether	1239	3.1	3.8	0.2
Thymol	1292	43.8	36.7	-
Carvacrol	1298	6.0	0.9	81.5
α-Copaene	1376	0.1	0.2	-
β-Caryophyllene	1420	1.0	1.9	1.7
β-Copaene	1429	-	0.1	-
α-Humulene	1456	0.1	0.2	-
*allo*-Aromadendrene	1461	-	0.1	-
γ-Muurolene	1477	0.3	0.3	-
Germacrene D	1478	-	1.0	-
Bicyclogermacrene	1496	-	0.2	-
α-Muurolene	1498	0.1	0.1	-
β-Bisabolene	1509	1.1	2.0	-
γ-Cadinene	1513	0.3	0.3	-
δ-Cadinene	1525	0.6	0.7	-
Spathulenol	1576	-	0.2	-
Caryophyllene oxide	1581	0.3	0.2	0.5
14-Hydroxy-9-*epi*-caryophyllene	1664	-	0.2	-
Monoterpene hydrocarbons		37.0	40.0	12.8
Oxygenated monoterpenes		56.5	47.8	82.4
Sesquiterpene hydrocarbons		3.6	7.1	1.7
Oxygenated sesquiterpenes		0.3	0.6	0.5
Non-terpene derivatives		2.2	2.9	2.6
Total identified (%)		99.5	98.4	100.0

^a^ Linear retentions index on a DB5 capillary column; ^b^ Not detected.

**Table 2:** Adjusted estimated marginal (EM) means of the mortality of *Calliphora vomitoria* adults exposed to the essential oils extracted from different oregano chemotypes.

Chemotype	Mean ^a^ ± SE	95% Confidence Interval	
		Lower Bound	Upper Bound
CC	73.447 ± 5.314 a	62.554	84.400
TCC	60.267 ± 5.314 b	49.334	71.190
TTC	88.041 ± 4.632 a	78.520	97.562

CC, carvacrol chemotype; TCC, thymol/*p*-cymene chemotype; TTC thymol/γ-terpinene chemotype. ^a^, Data are expressed as μL insect^−1^. Covariate (essential oils concentration) was evaluated at 0.375 μL insect^−1^. Different letters indicate significant difference (*p* < 0.05) by Bonferroni pairwise comparison.

**Table 3:** Toxicity by contact of three oregano chemotypes essential oils (EOs) to adults of *Calliphora vomitoria*.

EO	LD_50_^a^	95% CI ^b^	LD_95_^c^	95% CI	Intercept ± SE	*p*
CC	0.240	0.207–0.274	0.657	0.567 ± 0.787	2.331 ± 0.174	<0.001
TCC	0.312	0.274–0.351	0.854	0.736 ± 1.029	1.903 ± 0.152	<0.001
TTC	0.141	0.121–0.161	0.386	0.330 ± 0.465	3.203 ± 0.238	<0.001

^a^, Dose of EO that kills 50% of the insects; ^b^, confidence interval; ^c^, dose of EO that kills 95% of the insects. Data are calculated by Probit regression analysis and given as μL insect^−1^. CC, carvacrol chemotype; TCC, thymol/*p*-cymene chemotype; TTC thymol/γ-terpinene chemotype. Model slope = 3.76 ± 0.30; Pearson goodness-of-fit test, *χ*^2^ = 10.717, df = 7, *p* = 0.151; Parallelism test, df = 2, *p* = 0.648.

**Table 4:** Adjusted estimated marginal (EM) means of the mortality of *Calliphora vomitoria* eggs exposed to the essential oils extracted from different oregano chemotypes.

Chemotype	Mean ^a^ ± SE	95% Confidence Interval	
		Lower Bound	Upper Bound
CC	32.793 ± 2.769 a	27.271	38.314
TCC	60.525 ± 2.769 b	55.003	66.046
TTC	82.091 ± 2.769 c	76.569	87.612

CC, carvacrol chemotype; TCC, thymol/*p*-cymene chemotype; TTC thymol/γ-terpinene chemotype. Data are expressed as mean mortality percentage ± standard error. ^a^, Covariate (essential oils concentration) was evaluated at 0.025 μL cm^−2^. Different letters indicate a significant difference (*p* < 0.05) by Bonferroni pairwise comparison.

**Table 5:** Toxicity of three oregano chemotypes essential oils (EOs) to eggs of *Calliphora vomitoria*.

EO	LC_50_^a^	95% CI ^b^	LC_95_^c^	95% CI ^b^	Intercept ± SE	*p*
CC	0.038	0.023–0.063	0.235	0.122 ± 0.771	2.947 ± 0.128	<0.001
TCC	0.008	0.005–0.013	0.052	0.031 ± 0.125	4.300 ± 0.157	<0.001
TTC	0.013	0.008–0.020	0.080	0.046 ± 0.218	3.909 ± 0.151	<0.001

^a^, Concentration of EO that kills 50% of the eggs; ^b^, confidence interval; ^c^, concentration of EO that kills 95% of the eggs. Data are calculated by Probit regression analysis and given as μL cm^−2^. CC, carvacrol chemotype; TCC, thymol/*p*-cymene chemotype; TTC thymol/γ-terpinene chemotype. Model slope = 2.11 ± 0.08; Pearson goodness-of-fit test, *χ*^2^ = 142.76, df = 9, *p* < 0.001 (since *p* < 0.150, a heterogeneity factor was used in the calculation of confidence limits); Parallelism test, df = 2, *p* < 0.001.

## Data Availability

Datasets are available on request to corresponding authors.
